# Increasing Access to Places for Physical Activity Through a Joint Use Agreement: A Case Study in Urban Honolulu

**Published:** 2008-06-15

**Authors:** Jay Maddock, Lehua B Choy, Blythe Nett, Meghan D McGurk, Reid Tamashiro

**Affiliations:** Department of Public Health Sciences, University of Hawaii; University of Hawaii, Department of Public Health Sciences, Honolulu, Hawaii; University of Hawaii, Department of Public Health Sciences, Honolulu, Hawaii; City and County of Honolulu Department of Parks and Recreation, Honolulu, Hawaii; City and County of Honolulu Department of Parks and Recreation, Honolulu, Hawaii

## Abstract

**Background:**

To increase levels of physical activity (PA), interventions that create or enhance access to places for PA are recommended. Establishing a joint use agreement is one way to increase access to existing PA and recreational facilities. The purpose of this article is to present a case study of In-Motion, a pilot joint use agreement project at one urban high school in Honolulu, Hawaii.

**Context:**

Residents of urban Honolulu are underserved by the amount of parkland and recreational facilities available for their use. The Honolulu County Department of Parks and Recreation sought to implement a joint use agreement to use the facilities of one urban high school for a recreational program. The high school selected for the pilot project has a student population primarily from low-income and ethnic minority backgrounds.

**Methods:**

An assessment of the potential of 7 urban high schools to implement a joint use agreement was conducted to select the pilot site. In-Motion developed and implemented a joint use agreement. PA preferences of students, staff, and community members were assessed to guide recreational program offerings. Various recreational classes were offered free to the school community.

**Consequences:**

Several barriers to implementing the joint use agreement and recreational program were encountered. However, participants were satisfied with the recreational classes they attended and said that the In-Motion program helped them to engage in more PA. Program awareness by high school students and staff was high.

**Interpretation:**

In-Motion has successfully modeled a pilot joint use agreement and provided new opportunities for PA to the high school's students, teachers, and staff, and to community residents.

## Background

More than half of U.S. adults do not meet recommended physical activity (PA) levels (1) of at least 30 minutes of moderate-intensity PA on 5 or more days per week or at least 20 minutes of vigorous-intensity PA on 3 or more days per week (2). Additionally, many adolescents do not engage in sufficient amounts of PA (3). To increase PA levels among adults and adolescents, the *Guide to Community Preventive Services* recommends interventions that create or enhance access to places for PA combined with informational outreach activities (4). These interventions have the potential to result in a 25% increase in the number of people who exercise at least 3 times per week (5). The recommendation of the *Guide to Community Preventive Services* was made on the basis of a systematic review finding that the approach should be effective in diverse settings and populations (6).

Increasing access to safe places and opportunities for PA is particularly important for members of disadvantaged populations. Studies have documented decreased availability of opportunities for PA and of recreational facilities in low socioeconomic status (SES) neighborhoods and neighborhoods with high proportions of ethnic minorities (7,8). Reduced availability of facilities is associated with lower levels of PA and increased overweight among U.S. adolescents (8). In addition to a lack of available facilities, concerns about safety are another barrier to PA in underserved communities (9). Perceptions of poor environmental safety have been linked to decreased levels of PA. For example, teenagers who live in disadvantaged neighborhoods are less likely to live near a park that they consider safe and are less likely to be physically active than teenagers who live in more advantaged neighborhoods (10).

One means of increasing access to places for PA is to establish a joint use agreement (JUA). Broadly defined, a JUA is a policy that allows for shared use of facilities among partners. It formally outlines the terms and conditions of use, management, scheduling, maintenance, and liability, as well as the roles and responsibilities of partners (11). Advantages of a JUA may include cost sharing, limitations on liability, and improved access to recreational sites and opportunities for PA (12). Once implemented, a JUA can decrease barriers to community access and enhance use of existing facilities.

The purpose of this article is to present a case study of In-Motion, a JUA pilot project of the City and County of Honolulu Department of Parks and Recreation (DPR) implemented at Farrington High School (FHS) in Honolulu, Hawaii. In conjunction with developing a JUA, In-Motion offers a recreational PA program to students, staff, and community members, using school facilities both during and after school hours. The mission of In-Motion is to create opportunities for PA that are fun and that promote participants' confidence in their ability to be physically active.

## Context

In 2002, DPR sought to enter into an intergovernmental JUA with the Hawaii State Department of Education (DOE) to expand recreational activities for the youth and communities of urban Honolulu. Urban Honolulu has a shortage of community-based parkland coupled with a high population density. Compared with other areas of Honolulu County, urban Honolulu is underserved by the amount of DPR-maintained parkland and recreational facilities per capita resident population (13). The potential for expanding park space in urban Honolulu is limited by high land costs and lack of suitable sites. Increased access to public high school facilities within urban Honolulu would help to expand PA opportunities in many neighborhoods. However, partnerships between DPR and DOE at the high school level are uncommon. Therefore, a pilot project was undertaken to develop a model JUA and recreational program at one high school in urban Honolulu.

FHS, the school selected for the pilot JUA project, enrolls one of the largest student populations (over 2500 students) in the state and serves students primarily from lower SES and ethnic minority backgrounds (14). Immigrants who require instruction in English as a second language make up a large proportion of the student population. Over 60% of students receive free or reduced-cost lunches. The top 3 ethnic groups represented by students are Filipino (58%), Samoan (13%), and Native Hawaiian (12%) (14). All FHS students are required to fulfill 1.5 credits of physical education classes. However, only 24% of FHS students participate in organized, extracurricular school sports (13), and the PA levels of the remaining students are unknown. Furthermore, over half (64.3%) of adolescents living in the Farrington area reported living in unsafe neighborhoods (15), so providing a safe place for PA is important.

Located in the Kalihi neighborhood area, the FHS community is composed of over 46,000 residents (14). In general, the community mirrors the low-SES and ethnic minority backgrounds of the FHS students. The community has the highest percentages statewide of Filipinos (46.7%) and of foreign-born recent immigrants (15.6%) (15). Per-capita annual income is $14,634, which puts the community in the lowest quartile in the state. Residents of the community have higher rates of unemployment, higher use of welfare and food stamp assistance, and lower levels of home ownership than all residents in the state (15).

DPR received a grant from the Hawaii State Department of Health to implement the pilot JUA project. The grant was the primary source of funding, but additional financial support was provided by DPR and FHS. The project was approved by the University of Hawaii Committee on Human Studies. In-Motion was managed by 2 full-time project staff: a project manager (a DPR employee) and a project coordinator (a contracted employee).

## Methods

### Assessment of joint use potential

In 2004, DPR contracted an independent agency to assess the joint use potential of 7 urban Honolulu high schools (13). Data on available athletic facilities were collected and interviews with school principals and athletic directors were conducted to assess recreational needs and opportunities, use of athletic facilities after hours, and receptiveness to JUA participation. Farrington High School was selected for the pilot JUA project in August 2004 on the basis of the principal's receptiveness to establishing a pilot JUA project, active community involvement in the campus, and mutual benefits for DPR and FHS.

### Implementation of JUA

Once established at FHS, the pilot JUA project was named In-Motion. While the JUA was in development, In-Motion was able to conduct recreational activities. In January 2005, an initial JUA specific to FHS was drafted on the basis of an existing JUA developed in 1971 between DPR and DOE for the use of a baseball field. The JUA required approval from both DPR and DOE agencies, a multistep process involving individuals at different organizational levels. In the final step, the JUA was adopted by the Council of the City and County of Honolulu on June 7, 2006 (Resolution 06-159). Developing and officially approving the JUA took 18 months, and the support of the FHS administration was critical in enabling In-Motion to build its recreational program during this period.

### Assessment of PA preferences

To determine which physical activities In-Motion should offer, DPR partnered with the Healthy Hawaii Initiative Evaluation Team (HHIET), composed of researchers from the University of Hawaii, in developing surveys for FHS students, teachers, and staff and for residents of the community. The survey for community residents was translated into Samoan, Chuukese, Vietnamese, Tagalog, and Ilocano. Surveys were distributed in December 2004 to FHS students and staff, parents of FHS students, and community organizations and low-income housing developments in urban Honolulu. Incentives (e.g., water bottles, CDs) were given away during a raffle drawing to encourage participation. The surveys sought to determine which physical activities would be most popular, as well as the days and times that would encourage maximum participation in the In-Motion program.

Surveys were completed by 1385 FHS students, 112 FHS teachers and staff, and 64 community residents. Approximately 40% of respondents (41.2% of students, 40.3% of teachers and staff, and 39.7% of community residents) expressed interest in PA programs at FHS. The most convenient days and times for participation indicated were weekday afternoons, weekday evenings, and Saturdays. Students indicated that they wanted to attend PA classes during their school hours, especially during lunchtime. The most popular activities indicated were volleyball, strength training, and dance.

### Recreational program activities

The focus of In-Motion was providing organized recreational classes. Beginning in January 2005, In-Motion provided these classes to students, staff, and community members. Classes were advertised as "free and available to everyone," targeted beginning exercisers, did not have established routines, and were designed to provide a safe and fun environment to exercise. Activities were chosen on the basis of identified priorities, availability of instructors, availability of facilities, availability of existing activities in the community, suggestions from FHS administration, and student feedback. Depending on the number and type of recreational program offerings, approximately 6 to 8 instructors were hired for each program session.

Teen classes included hip-hop, salsa, and swing dance; volleyball; learn-to-swim; circuit training; walking; hula; strength training; capoeira (a Brazilian martial art, combining dance and fighting techniques); weight-loss support group; and Physical Fridays (physical activities and health lessons for a selected group of high-risk students every Friday). Activities like capoeira, salsa dance, and volleyball were offered during lunchtime when students were free. In-Motion staff organized dance competitions and conducted volleyball tournaments for students. Field trips for off-campus recreational activities (e.g., ice skating, laser tag) were offered as incentives to students who logged time in the walking program.

Adult classes were offered with different target groups in mind. Aerobics, yoga, and group exercise were offered during the early afternoon to target the FHS teachers and staff. Basic body fitness; hip-hop, salsa, and swing dance; and strength training were offered during the early evening to target community members who were on their way home from work. The water exercise class was offered to senior citizens during early weekday mornings. The walking program offered a window of time for any adults in the community to come walk on campus.

### Participant recruitment

Recruitment of participants for classes depended on the type of class and its target population. Teenagers were recruited through school publicity (i.e., daily bulletins, morning announcements, banners, and flyers), physical education classes, and campus activities such as registration tables, lunchtime activities, social clubs, and performance demonstrations. Recruitment was also conducted at community organizations and middle schools.

Adult participants were more difficult to recruit. Through a communications strategy developed by a contracted marketing company, multiple means of disseminating program information were identified. These means included radio-based public service announcements, newspaper advertisements, and flyers distributed throughout the community.

### Project evaluation

HHIET developed an evaluation plan for In-Motion on the basis of the Centers for Disease Control and Prevention's *Framework for Program Evaluation in Public Health* (16). Process evaluation was conducted for the JUA, and several surveys were developed to assess participant outcomes and program awareness and impact. Participant surveys were distributed at the end of each recreational class to measure participant satisfaction and perceptions about PA. In May 2006, students, teachers and staff, and community members were surveyed to determine program awareness and impact. These surveys were distributed to all homeroom classes, all mailboxes of teachers and staff, and 3 community organizations. Incentives (e.g., gift cards) were used to encourage student, teacher, and staff participation in the surveys.

## Consequences

### Establishing the JUA

The JUA set parameters for use and maintenance of facilities; fee schedule; staffing; use of materials and equipment; liability; and risk of loss. DPR assumed liability for In-Motion activities and responsibility for supervising and managing In-Motion activities. The school (DOE) assumed responsibility for general cleaning and maintenance of the facilities and did not charge DPR any fees for use of facilities.

Lack of writing expertise was an initial barrier to creating the JUA, and obtaining approval for the JUA required persistence. Once the document was written, the primary challenge was moving it through several bureaucracies in a timely manner. The JUA passed through many hands before it was heard by the City and County Health and Safety Committee and the full Council. This phase proceeded slowly, because both groups met only monthly. The status of the JUA was checked almost weekly, and without this shepherding, completion would have taken much longer. Although both agencies generally agreed on issues of liability and facility maintenance, reservations over the specific language of the document and delays in passing it among the many decision makers contributed to the lengthy process.

### Program implementation

Successful program implementation can be attributed to several factors. First, the primary key to success was the trust and effective communication established among key players, including the FHS principal and faculty and DPR project staff. Second, FHS administration, particularly the principal, were accommodating, providing office space on campus, communication systems, storage space for equipment, and administrative assistance. Third, In-Motion consistently adapted the programs in response to participant and instructor feedback. Fourth, In-Motion offered the recreational classes for free, thus eliminating one barrier for low-income families.

Overcoming other barriers facing people from low-income and immigrant populations was difficult. For example, some parents did not speak or write in English well enough to complete permission forms, and some family members lacked transportation to FHS.

Finding qualified staff available to teach activities during the day for a few hours per week also proved to be difficult; however, In-Motion was able to recruit several skilled instructors who could attract and retain participants. Recruiting teenagers to stay after school was another challenge, because many had other activities (e.g., jobs, clubs, family responsibilities) or preferred to spend time with friends. However, once participants joined In-Motion, their word-of-mouth publicity was a powerful source of program recruitment and was crucial to garnering program awareness.

### Participant outcomes

Since project inception, In-Motion has served more than 1000 registered participants and held over 900 class sessions. As of December 2006, surveys were collected from 320 participants (participants from spring 2005 classes and lunchtime activities were not surveyed). More than 90% of participants present on the last day of class completed a survey; however, the overall response rate accounting for all registered participants was only 34%.

Most participants were female (66.2%), younger than 18 years (52.8%), Filipino (40.9%), and FHS students (52.2%) (Table 1). Most participants were satisfied with the recreational class that they attended and said that In-Motion had provided them with a safe place to exercise, motivation to exercise more, and a feeling of confidence that they could exercise 30 minutes per day on most days of the week (Table 2).

### Program awareness and impact

After In-Motion recreational classes had been offered for 1 year, surveys were distributed to FHS students, teachers, and staff, and to community members to assess program awareness and impact. A total of 906 students (36% response rate) completed surveys. When asked whether they had heard of In-Motion, 66% (n = 599) of students indicated that they had. Of the students who were aware of In-Motion, 82% (n = 493) also knew that its classes were free and available to the public. Approximately 11% (n = 98) of responding students indicated that they had attended one of In-Motion's classes; however, this proportion is probably higher, because students may not have realized that some classes they attended were affiliated with In-Motion.

Of the 260 surveys distributed to teacher and staff mailboxes, 68 (26% response rate) were completed and returned. Of respondents, 99% (n = 67) had heard of In-Motion, and 97% (n = 66) knew that classes were free and available to the public. All 68 respondents agreed that the program had a positive impact on the school and that it was beneficial to students. They reported that the program provided needed opportunities for PA, incurred social benefits (e.g., making new friends), kept students out of trouble, and promoted healthy lifestyles. Approximately 90% (n = 62) said that In-Motion did not create extra work for them, and 99% (n = 67) indicated a desire to continue In-Motion at FHS.

A convenience sample of 66 community members completed the survey about In-Motion. Overall, 53% (n = 35) had heard of In-Motion; of those respondents, 83% (n = 29) knew that the classes were free and available to the public. Of the 66 respondents, 14 (21%) had attended one of In-Motion's recreational classes.

## Interpretation

In-Motion has successfully modeled and implemented a pilot JUA between DPR and DOE. The JUA for FHS can be adapted by other schools to increase opportunities for PA. For DPR, the existing JUA at FHS makes establishing similar partnerships with other schools easier. Recreation staff and school administrators would not only be able to adapt the JUA but would also have their reluctance toward entering into such a relationship allayed by the success of In-Motion. Having a JUA added legitimacy to the project and signaled a higher level of commitment to it. Despite the challenges to implementing a JUA, the agreement helped to overcome barriers — such as safety, insurance costs, and liability concerns — associated with making school facilities available to the public (17).

However, although the JUA formalizes the relationship between DPR and the school and may lead to systemic changes that increase PA, other factors are critical to the short-term success of In-Motion. These factors include the ability of key players (i.e., school principal and faculty and DPR project staff) to work well together, respect and trust one another, and be responsive and adaptable to inevitable setbacks. Holding recreational activities during school hours (e.g., lunchtime volleyball) allowed In-Motion staff to develop relationships with students, which was critical to increasing participation in after-school recreational activities. Furthermore, despite the publicity, attracting participants was mainly accomplished through word-of-mouth. To reach the adult community members, allowing time for word-of-mouth to spread should be expected. Some limitations of our study were the low response rate for some of the surveys and the potential survey response bias that may have occurred if people who liked the program were more likely to participate.

DPR is exploring options for continuing In-Motion once the current funding period is completed, including expansion to other schools and community organizations. However, no other schools or communities have chosen to adapt the JUA implemented by In-Motion at FHS. The JUA is a sustainable policy that only requires In-Motion to schedule facility use for future activities at FHS. However, the recreational activities offered by In-Motion depend on external funding, and they will continue only if such funding is secured. In-Motion has provided new opportunities for PA in a safe environment for students, teachers and staff, and community members. The project can serve as a model for other JUA partnerships to increase PA in underserved communities.

## Acknowledgments

In-Motion received funding from the Tobacco Settlement Special Fund through a grant from the Healthy Hawaii Initiative, Hawaii State Department of Health. Additional financial support was provided by the City and County of Honolulu DPR and FHS. We acknowledge the work of Nalani Aki, Carol Matsuoka, Ronn Nozoe, Linda Fujihara, Jayson Chun, Joyce Mitsunaga, and PlanPacific, Inc. We thank Principal Catherine Payne and the students, teachers, and staff at FHS, and members of the Kalihi community for their support.

## Figures and Tables

**Table 1:** Participant (N = 320) Characteristics, In-Motion Recreational Classes, Honolulu, Hawaii, June 2005 – December 2006

**Characteristic**	No. of Participants (%)
**Sex**	
Female	212 (66.2)
Male	99 (30.9)
Missing data	9 (2.8)
**Age, y**	
<18	169 (52.8)
18-35	30 (9.4)
36-55	41 (12.8)
≥56	76 (23.8)
Missing data	4 (1.2)
**Race/ethnicity**	
Chinese	30 (9.4)
Filipino	131 (40.9)
Japanese	35 (10.9)
Native Hawaiian	32 (10.0)
Samoan	20 (6.2)
White	19 (5.9)
Other	46 (14.4)
Missing data	7 (2.2)
**Type of participant**	
FHS student	167 (52.2)
FHS teacher/staff	14 (4.4)
Community resident^a^	90 (28.1)
Noncommunity resident^a^	43 (13.4)
Missing data	6 (1.9)

FHS indicates Farrington High School.

a Resident of the Kalihi neighborhood in urban Honolulu.

**Table 2:** Participant (N = 320) Outcomes, In-Motion Recreational Classes Survey, Honolulu, Hawaii, June 2005 – December 2006

**Outcome**	No. Who Strongly Agree (%)	No. Who Agree (%)	No. Who Are Neutral (%)	No. Who Disagree (%)	No. Who Strongly Disagree (%)	No. With Missing Data (%)
I am satisfied with this class.	240 (75.0)	55 (17.2)	14 (4.4)	4 (1.2)	5 (1.6)	2 (0.6)
I am confident that I can exercise 30 minutes/day on most days of the week.	167 (52.2)	82 (25.6)	47 (14.7)	17 (5.3)	4 (1.2)	3 (0.9)
I have a safe place to exercise.	197 (61.6)	71 (22.2)	37 (11.6)	7 (2.2)	7 (2.2)	1 (0.3)
The In-Motion recreational classes have helped me to exercise more.	190 (59.4)	75 (23.4)	35 (10.9)	6 (1.9)	3 (0.9)	11 (3.4)
